# Some Reflections on Psychotherapy

**Published:** 1908-10

**Authors:** Julius Grinker

**Affiliations:** Chicago, Professor of Nervous and Mental Diseases, Chicago Postgraduate Medical School; Assistant Professor Clinical Neurology, Northwestern University Medical School; Consulting Neurologist to Cook County Insane Hospital and Infirmary


### Original Articles.

For Texas Medical Journal.

### Some Reflections on Psychotherapy.

BY DR. JULIUS GRINKER, CHICAGO,

Professor of Nervous and Mental Diseases, Chicago Postgraduate Medical School; Assistant Professor Clinical Neurology, Northwestern University Medical School; Consulting Neurologist to Cook County Insane Hospital and Infirmary.

Long before modern medicine was born psychotherapy had already accomplished wonders. When scientific medicine did arrive, mental therapy had to change its garb and was even ostracised for a time. Today it is firmly established upon a solid foundation and, in my opinion, not until human beings cease to have minds will mental treatment be displaced from its well-earned position.

Every physician, be he a country practitioner or a fashionable city doctor, consciously or unconsciously practices mental medicine. The stronger the physician himself is convinced of the efficacy of his treatment, the more effective, as a rule, are his remedies. Often it is not medicine that the patient needs, but rather a good dose of "doctor," as one of my friends expressed it. In short, the physician's personality may count for more than his prescription. This fact seems to be well known, even to the medical student, who in the latter part of his course begins to prepare himself for practice by raising a crop of hair on his chin. After he has left the medical school you may find him, if he is sensible, with full beard, silk hat, long coat, a professional smile and smooth vocabulary, sitting in a well-equipped office containing more instruments and electrical appliances than he can possibly use in the coming ten or more years of his practice. If, perchance, he thinks you are a patient, he will admit you with a most self-possessed and confidence-inspiring manner, which seems to say: "I am ready for any emergency, from a toothache to a gastro-enterostomy; have no fear to entrust yourself to me, sir." Why does this country-bred boy so change his dress, manner and even his speech? Because he wishes to make a mental impression on his patient, or for short, he practices psychotherapy. And, though the practice is as old as the world, the theory of it, that is, the conscious recognition of its phenomena, conditions, causes, prevalence and importance in medical practice, is comparatively new.

Some men are born psychotherapeutists, and, consequently, there is no need for such to learn the art. Their mere entrance into the sick room brings light, life and hope. Emerson probably had an individual of this type in view when he wrote: "It costs a beautiful person no exertion to paint her image on our eyes, yet how splendid is that benefit! It costs no more for a wise soul to convey his quality to other men. And everyone can do his best thing easiest. He is great who is what he is from nature and who never reminds us of others. But he must be related to us, and our life receive from him some promise or explanation. I can not tell what I would know, but I have observed there are persons who, in their character and actions, answer questions which I have not skill to put."

While but few are so fortunate as to inherit a great personality, many can acquire the outward signs and characteristics of greatness. The theme of conscious and well-directed psychotherapy in its present aspect has already been before the profession many years, yet we have allowed quacks and healers to grasp the essentials and to apply them in practice, while we supinely revenge ourselves by calling them "humbugs." That there are still those among us who persistently refuse recognition to mental methods in treatment is a sad commentary upon the human understanding. Nothing would seem more self-evident than that psychotherapy is part of general therapy, and belongs exclusively to the domain of the legitimate practitioner, and yet we have in our ranks men who have sunk to the level of "apologists" and lackeys of "religious" healers. Evidently it is easier—but in my opinion far less dignified—to send a patient to a clergyman for treatment than to master the principles of psychotherapy and how to apply them. These men not only lay bare their own weakness, but they mistake personal incompetence for that of the entire profession by ignoring the fact that there are many physicians who practice mental in conjunction with other forms of therapy.

What is psychotherapy? In a general way, it signifies the use of any means whatever for the purpose of so affecting a patient's mentality as to produce amelioration or removal of symptoms. The effects are never produced by chemical or mechanical agents, but the latter may serve as convenient vehicles. One great distinction between psychic and drug treatment is that the latter often exerts its effects upon metabolism without the patient's co-operation and sometimes even against his will or expectations, the former always presupposes the patient's active or passive participation in altering his mode of thought in the direction of cure.

There are two principal methods by which patients are psychically influenced for purposes of treatment:

1. By calling into service more or less mysterious agencies, that is, by altering the patient's consciousness may making him an obedient tool of the physician's will.

2. Subsequent to a correct analysis of the symptoms the physician endeavors by methodic instruction and persuasion to srtengthen the patient's will and his powers of resistance in order to enable him to direct his thoughts into healthy channels, thereby producing beneficial results.

The first-mentioned method, until recently the more popular and better known, is the so-called treatment by suggestion.

The Century Dictionary defines suggestion thus: "The insinuation of a belief or impulse into the mind of a subject by any means, as by words or gestures, usually by emphatic declaration." Not every influence exerted by one man upon another is suggestion, but only when an individual accepts an idea without adequate physiological excitation which he then transforms into an act, idea or sensation.

According to Forel, "Suggestion is the production of a dynamic change in the nervous system of an individual by arousing in his mind the idea that such change is either taking place, has already occurred, or is about to be realized. Verbal suggestion is the process by which ideas are implanted into the mind through words. Suggestibility is that quality of mind which enables one to accept suggestions. It is generally understood that most people are suggestible; some can be influenced in the waking state and others must be placed into the state of passivity called hypnotism, before they can accept suggestions. Hypnotism itself is considered suggestion carried to the extreme. It is beyond the scope of this paper to discuss the technique and practical application of hypnotism. Many excellent books have been written on the subject and the practice can be easily acquired by anyone who wishes.

Beside psychotherapeutists have of late learned to rely almost exclusively upon suggestion and persuasion in the waking state, and, consequently, hypnotism is being gradually relegated into the background. In speaking of suggestion it must be stated that an individual may consciously or unconsciously produce suggestions within himself without the aid of another person, in which case he is the subject of autosuggestions. There are beneficial and harmful suggestions and autosuggestions. Suggestions influence a person's acts intuitively, blindly—not by arguments. Indeed, reason normally offers an effective barrier against unhealthy suggestions, which latter may not inaptly be compared to toxines generated within (autosuggestions) or without the body (heterosuggestions), while reason itself is built on the basis of firmly organized brain automatons, and may be compared either to phagocytes or body protectors. Proper counter-suggestions may be likened to antitoxines or vaccines, which, when introduced into the mind, neutralize the effects of unhealthy suggestions, gradually permitting reason to again assume full control. Suggestive treatment is administered in several ways: Of the physician's personality I have already spoken; but, more than this, his statements to the patient must tend to assuage fear and ought never to admit of doubtful interpretation. Every word is to convey to the patient a hope or promise of cure. If medicines are prescribed, they must not be given to the patient as one would give them to a horse, but positive assurance of relief should always accompany them. Not all individuals will need the same amount of medicine, nor will all need the same quantity of suggestion. "One must learn to distinguish those who need an ounce of medicine and a grain of wisdom from those who require an ounce of wisdom and a grain of medicine," as Schofield so aptly puts it. Dr. Weir Mitchell correctly points out that the doctor who gives much medicine and many medicines, who is continually changing them, and who does not insist with care on knowing all about the patient's habits as to diet, meal times, sleep, modes of work and hours of recreation is, on the whole, one to be avoided." One rule in mental therapeutics never to be forgotten is that the cure of the sick body or exhausted nerves must precede the cure of the sick mind; in other words, the physical must precede the psychical.

After having thoroughly penetrated the patient's mental machinery, one will not be at a loss for proper suggestions. Furthermore, it is as difficult to give specific rules for mental therapy as it is to mention specifics for physical disease. Although we are still without a mental opsonic index, we can learn with a little effort to supplant unhealthy suggestions with healthy ones.

I have now reached the second and, in my opinion, the most important part of our subject, namely *psychotherapy by educational methods.* This method consists in giving the patient a logical and truthful explanation of his symptoms and how they originated; it teaches him to adopt such means as will strengthen his will. It is needless to state that children and those who have the minds of children are not subjects for such treatment. Appeals to reason, explanations of right living and thinking are the principal weapons used. One successful effort is utilized as a stimulus to still greater efforts. The patient is spurned on to do his best, not by force from without, but by the exercise of powers from within; in other words, he is induced to act as a free being and not as a puppet in the hands of an operator. This is truly educational treatment, for, according to Emerson, "Man is endogenous and education is unfolding. The aid we have from others is mechanical, compared with the discoveries of nature in us. What is thus learned is delightful in the doing, and the effect remains. Right ethics are central and go from within outwards. Gift is contrary to the law of the universe." Attention and interest must be aroused in the patient, just as in the student, not by constant admonition to be good, but by primarily furnishing examples for imitation. If he lives in a faulty environment, this must be changed, or all effort will be in vain. Good companions, preferably of the same age as the patient, will exert a greater influence on him than all the preaching in the world. Let us again quote the New England sage, who says, "We are all wise in capacity, though so few in energy. There needs but one wise man in a company and all are wise, so rapid is the contagion. Even normally we can not escape our environment, our class, our city. There are vices and follies incident to whole populations and ages; men resemble their contemporaries even more than their progenitors. It is observed in old couples, or in persons who have been housemates for a course of years, that they grow alike and if they should live long enough we should not be able to know them apart." It is within the knowledge of all that the contact of nervous patients with each other has a tendency to multiply symptoms in them. The worst company for a nervous daughter is her neurotic mother. Perhaps the greatest benefit from the Weir Mitchell rest cure, which enforces isolation from friends and relatives and compels association with a strange, sensible nurse, is through the necessary change of environment. There are many ways of changing environment beside sending to a sanitarium.

Next to the careful selection of proper associates comes a wise choice of your patient's daily reading. Only books and magazines that are stimulating, refreshing and overflowing with optimism should be recommended to the patient, while all yellow journals, the chroniclers of crime and monstrosity, should be banished from his sight.

No less important is the inculcation of the idea of methodic work suitable to the individual's temperament. Work is the most powerful weapon we have against the habit of introspection which latter is usually the result of indolence. Many a woman who for months has not raised a finger in her household, suddenly discovers that she can do without help after the physician has explained to her that regular exercise strengthens the organism. We have ceased to send every nervous woman to bed, ostensibly to take a rest cure, but really to fill her mind with all sorts of phobias. Instead we order the so-called work cure. And not only do we aim to occupy their bodies, but we also plan to arouse their slumbering intellects. We appeal to their moral sense and arouse their interest in charity and settlement work. "Serving others is serving us" is a good text for preachment. I once heard a liberal clergyman in this city say to the women of his church, "When you do charity you receive infinitely more than you give." This statement strikes me as especially applicable to the indolent neurotic, whose empty life is often through charitable work converted into one of absorbing activities. These are only a few of the things in which patients can become interested. There are many others that will readily suggest themselves to the thinking physician.

Now some of my friends will contend that the methods as outlined may very properly be advised, but will the patient agree to follow them? My answer is that the educational methods of psychic treatment do not differ radically from other educational lines, in which we have to resort to the various motives governing human actions; among these are rewards, threats, punishment, even flattery.

Reward may take the form of granting a patient's request, provided he complies with your conditions. The hope of reward exercises an immense influence not only upon children, but also upon adults.

Threats are occasionally necessary. The patient is merely warned that certain privileges will be curtailed if your directions are not followed.

Punishment may become necessary, but should never appear in the form of revenge, otherwise resentment will follow. I imagine that the old-time treatment of hysteria by asafoetida and valerianates was a kind of punishment, and it must be admitted that it often did abbreviate hysterical attacks. But there are better methods than the administration of malodorous medicines. For instance, a sensitive patient who has always received courteous treatment from her physician will be set to thinking when he administers a rebuke by a short, gruff greeting or by studied indifference.

In still other cases, a joke or a bit of flattery will arouse a patient's latent energies. Tell a certain indolent person who is averse to taking the prescribed walk: "In my opinion you belong to the active, energetic type; walking will be mere child's play for you," and he will run, not walk.

We must always inspire our patients with hope of recovery; sometimes it is well to relate similar cases in which we accomplished cures. At no time should we drop any hint which the patient may interpret as an unfavorable prognosis, nor should we pay undue attention to his local complaints, which we know to be only subjective.

Occasionally a remark made to a tattling nurse or a friend will produce results when a direct statement to the patient may fall on barren soil. Seeing how necessary it is for the physician to study the devious paths of his patient's mind and the most advantageous points of attack, I can not comprehend how frequent contact of physician and patient can be harmful according to the opinion of a Boston physician. We will again let his distinguished townsman, Emerson, answer him: "Talk much with any man of vigorous mind, and we acquire very fast the habit of looking at things in the same light, and on each occurrence we anticipate his thought." Personally, I think repeated conversations with sane doctors, that is, with those who do not learn their psychotherapy from Christian Scientists, Christian Psychologists or other "healers," can do no harm to patients and must do them a great deal of good.

In what diseases will psychotherapy do good? In all or nearly all. In functional nervous diseases it will do most good. Even in organic disease, symptoms may be relieved and can even be made to disappear by properly directed psychic treatment.

As this paper deals only with the general aspects of this large subject, details can not be entered into and illustrative case histories can not be cited.

*Summary and Conclusion.*—1. There are two methods in psychotherapy, (a) suggestion, (b) education and persuasion.

2. Hypnotism, a form of suggestion, should be employed as a last resort, but it has its legitimate uses.

3. Psychotherapy is part of general medicine and surgery, consequently it should be utilized by physicians and not left to quacks or healers.

4. A most painstaking physical and mental examination is a necessary prerequisite to successful psychic treatment, as the patient must often be treated both physically and psychically.

5. Psychotherapy should be incorporated in the regular curriculum of every medical school, because the best interests of the public and the profession demand that this powerful weapon for good, if properly used, shall be placed early into the hands of sane and safe men.

